# 

**Published:** 1872-02

**Authors:** 


### Contents of February Number.

### ORIGINAL DEPARTMENT.

ART. I.—Medical Society of the County of Albany, Semi-Monthly Meeting, Jan. 11th and 24th, 1872. Reported by James S. Bailey, M. D.. 241

ART. II.—On the Recent Advances in the Theory of Vision. By H. Helmholtz. Translated by F. W. Abbott, M. D.... 245

ART. III.—On the employment of Taxis in Strangulated Hernia. Read at the regular meeting of the Medical Association. By C. C. F. Gay, M. D.... 256

ART. IV.—Clinical Remarks on Diabetes, and on Delirium Tremens. By Prof. Thos. F. Rochester, M. D. In the Hospital of the Sisters of Charity. Reported by Edward N. Brush, member of the class .... 264

ART. V.—Case of Fracture of the Skull. By Charles W. Saunders, M. D. Belfast, N. Y.... 268

### EDITORIAL DEPARTMENT.

Medical Society of the State of New York.... 269

Annual Commencement of the Medical Department of the University of Buffalo.... 271

Rush Medical College .... 273

A New Work by the late Prof. Dunglison.... 273

New Medical Journal.... 273

The National Medical Journal.... 274

Vermont Medical Journal.... 274

BOOKS REVIEWED:

The Principles and Practice of Surgery. By John Ashhurst, M. D. Review by. C. H. Richmond, M. D., Syracuse, N. Y. 274

A Clinical Manual of Diseases of the Ear. By Lawrence Turnbull, M. D.... 280

